# American Dental Patents

**Published:** 1853-07

**Authors:** Geo. W. Kendall

**Affiliations:** Cincinnati


### Editorial.

---

AMERICAN DENTAL PATENTS. BY GEO. W. KENDALL, CINCINNATI.

About a year since, Mr. Kendall gave us a history of Dental patents, and now in the April number of the American Journal of Dental Science, we have this history continued, and so far as it is a correct history, we are glad to see it. It is important that history be correct in all particulars, and that this may be the case, historians should be unprejudiced, and state transactions in all their truthful reality. They should also avail themselves of the best public documents by which to arrive at facts. In these particulars, we fear Mr. Kendall has sadly departed from his duty. In the continued historical sketch which Mr. Kendall gives in the last number of the Journal, there are two or three very unjust and gross errors, which aim a blow at the "Mississippi Valley Association of Dental Surgeons," and also at the Ohio College of Dental Surgery, and at the Faculty thereof, which justice requires should be corrected. In so far as this attack implicates ourself, we care but little. We hope to survive it. We shall therefore merely make the corrections, and not attempt to answer Mr. Kendall's "unanswerable arguments."

The first error is in relation to the action of the Mississippi Associations of Dental Surgeons. He remarks that "as an evidence of the upward progress of opinion, in reference to the patent system in the Profession, we may refer to the resolution passed by the Mississippi Valley Association, at its last meeting, declaring it derogatory to the professional character to patent improvements, the same having been lost by an overwhelming vote at the previous meeting." Now let us look at the facts; at the meeting preceding the last. The following resolution was adopted.

"*Resolved*, That it is now and always has been the sentiment of this society, that it is derogatory to the Professional character of any of its members to patent any dental instrument or mode of practice."

This is the resolution which Mr. Kendall says, was *lost* by an "overwhelming vote."

Let us see now what was done at the last meeting. By reference to the published minutes to which we suppose Mr. Kendall had access. We find the following resolution was offered.

*Resolved,* "That a committee of three be appointed to take into consideration the course of Dr. Allen, on the subject of patents, and report to-morrow."

"The question being taken on the adoption of the above, *it was lost,* because of the time it would take, and which could be more profitably employed, and as Dr. Allen had been already heard, in vindication of himself."

This is the only resolution offered at the last meeting having any bearing on the subject, and this was lost because the society thought it could spend the time more profitably. The society as a body, does not feel that it is necessary to be continually harping on this subject. Let us now see the evidence of an "upward progress of opinion" as it regards the society on this subject. We go back to the second annual meeting and get the first action on this subject. It is as follows:

*Resolved,* "That viewing ours as an important branch of one of the liberal professions, we feel bound to disapprove of any member of this society, patenting any instrument or mode of practice pertaining to our profession, and that further action on this subject be deferred, until our next annual meeting." Adopted.

Here we find the same disapprobation expressed, and we believe the time never has been, when the same views have not been entertained by a majority of the society, and the Society never has refused to meet the question when brought in a proper shape before it; although a few of us have always been opposed to applying it in that sweeping sense expressed in the resolutions; but were willing that a "piece of mechanicism applied to the arts," and which could be used without any "injury to the public," which should not interfere with the "ameliorations of the ills of life, that such might (even by a professional man) with propriety be patented." We have given, however, the action of the Society on this subjct, and are willing to abide by its decissions.

Mr. Kendall after having unburdened himself in relation to the society, proceeds to say,

"May we not therefore indulge the hope, that ere many years roll around, that instead of a majority of the Professors in the 'Ohio College of Dental Surgery' advocating by precept or practice the propriety of patenting professional improvements, they will all be united in pointing out to the pupils under their charge, the *true* path of the Professional gentleman."

Here the majority are represented, as advocating by "practice or precept the patenting professional improvements." The truth is so far as we know, but one of the Faculty have ever taken out or attempted to take out a patent, this is Dr. Allen. As for ourself, we have never by precept advocated the patenting of other than mechanical articles, and these only so far as they may not Professionally interfere in the treatment of disease. We do not consider Dr. Allen and ourself as a majority of the Faculty—as it regards however the action of the Faculty on this subject, it has been entirely of a different character.

When Dr. Allen received his appointment, which was before his patent for continuous gum was taken out. There was a perfect understanding that the class should be taught every thing in his department—that no secrets should be withheld from the students. That as teachers we conceived it to be our duty to give the students who came to us for instruction, all the information on the different departments we taught, which was in our power and without any other charge than the regular tuition fee. Dr. Allen's views coincided with ours on this subject, and he has ever acted on this principle, and has with a zeal and energy which is highly creditable applied himself to the advancement of the student. Mr. Kendall is certainly not ignorant of the fact that Dr. Van Emon has never been a Professor in the school. He was for two years demonstrator of mechanical Dentistry, and resigned this post last spring.

But Mr. Kendall, not content with the representation which he has given, goes on to say, that "it has been urged by our worthy Professors, that if Dentists were not allowed to patent improvements they might invent or discover, there would be no such improvements announced, as no one would labor to effect them, unless he could be *paid for it.*" Here he still uses the plural (Professors) as if this was the opinion of a majority of them, and still not content with this; but to fasten it little more directly on us, he makes a quotation from an article of ours on this subject. That the "disposition to patent every little improvement in instruments or appliances for the labaratory is illiberal." The latter opinion we expressed. Prof. Allen, we believe, is willing to acknowledge the former, and we believe he is the only one of the Faculty that has ever expressed them.

There is only one other statement made in Mr. Kendall's history, which we shall at present notice. Facts in history when mixed up with much which is false, need all the testimony that can be adduced to give them credence. Mr. Kendall gives the following as a fact in his history. "I who am nothing—a nobody—not even an honorary graduate of the Ohio College of Dental Surgery!" Now we can assure the Profession, that so far as the honorary degree is concerned, this statement can be relied on. The records of the Institution confirm this so far at least as silence on the subject is concerned. They only make known the fact that Mr. Kendall is a matriculated student of the school, and has been entitled to one course of instruction in the institution. The record does not say, that which we believe is the truth; that he is the only individual who has ever been entitled to this as a compliment to himself or friends.

If Mr. Kendall wishes to take a tilt at the patent system, he can do it just as genteelly some other way, as by making an invidious attack on the Mississippi Association of Dental Surgeons, or the Ohio College either. Prof. Allen's views are not "hid under a bushel," and if he wishes to break a lance with him, the Drs. armor is on, and has oft received the full force of his opponents charge. As for ourself, not being capable of presenting any new light on the subject, we supposed we were not to be "further noticed." But not wishing to complain, we would say in the chivalrous language of Knight errandry, "On, Stanley on."

